# Liraglutide Improves the Survival of INS-1 Cells by Promoting Macroautophagy

**DOI:** 10.5812/ijem.8088

**Published:** 2013-07-01

**Authors:** Jia jing Yin, Yan bo Li, Ming ming Cao, Yang Wang

**Affiliations:** 1 The Department of Endocrinology, The First Affiliated Hospital of Harbin Medical University, Harbin, China

**Keywords:** Liraglutide, Autophagy, Type 2 Diabetes, Free Fatty Acid, INS-1cells

## Abstract

**Background:**

Type 2 diabetes mellitus (T2D) is a metabolic disease characterized by dysfunction of pancreatic beta cell and insulin resistance. Liraglutide, which has many special anti-diabetes biological effects, is found to inhibit beta cell death and ameliorate endoplasmic reticulum stress (ERs) induced by free fatty acid (FFA). Macroautophagy (hereafter referred to as autophagy) altered by FFA is also associated with the dysfunction or death of pancreatic beta cells.

**Objectives:**

We aim at proving that Liraglutide improves the survival of INS-1 cells by promoting autophagy.

**Materials and Methods:**

Cell survival was assessed by CCK8 assay. The percentage of apoptotic cells was determined by flow cytometric assay after Annexin V-FITC/PI staining. Expression of LC3 was detected by western blotting. MDC staining and transmission electron microscopy (TEM) were used in the measurement of autophagy.

**Results:**

Apoptosis induced by PA in INS-1 cells was significantly resolved after Liraglutide treatment. Simultaneously, autophagy was enhanced with the treatment of PA and Liraglutide. Conclusions: Liraglutide appears to protect INS-1 cells from apoptosis FFA-induced by promoting autophagy.

**Conclusions:**

These findings provide a novel role for GLP-1 analogue in preventing or treating with T2D.

## 1. Background 

Type 2 diabetes mellitus (T2D), as a metabolic disease, is characterized by dysfunction of pancreatic β cells and insulin resistance. In recent decades, with the increasing prevalence of T2D, western diets which compose of both saturated fatty acids (FFAs) and trans-saturated fatty acid have been decided as the environmental factors contributed to the pathogenesis of diabetes. Glucolipotoxicity has been regarded as the key point contributed to the increasing β cell apoptosis rates and progressive β cell loss in T2D (1). Thus, we focus on the development of ways to protect β cell from apoptosis induced by FFA and the treatment strategies improving β cells function. 

Glucagon-like peptide-1(GLP-1), an incretin released from the L-cells of the small intestine, targets pancreatic β cells to release insulin and reduce glucagons production in response to food intake (2). In addition, GLP-1 also possesses some special anti-diabetes biological effects, such as anti-apoptosis, improving cell proliferation and differentiation (3-5). Liraglutide is a human GLP-1 analog with 97% amino acid homology to native human GLP-1 (6), and its protective actions against diabetes are mediated at the level of the β cell, as well as in peripheral tissues. Treating with Liraglutide subsequently after American lifestyle-induced obesity syndrome(ALIOS) diet shows a marked reduction in the lipid load in hepatocytes (7). It is found that hyperinsulinemia and insulin resistance caused by high fat diet suppress autophagy. The mechanism of FFA-mediated autophagy is still unclear. Researches demonstrated that high insulin production induced by elevated FFA in β cells overwhelmed endoplasmic reticulum (ER) folding capacity and unfolded protein response (UPR), which finally resulted in endoplasmic reticulum stress (ERs). Autophagy, acting as a degradation system, may be responsible for removing the overload of unfolded or misfolded protein that exceeds the ER capacity and contributes to the ameliorate of ERs. The ER-selective UPR induces reticulophagy, which may serve to reduce the volume of ER and unfolded ER proteins (8). Singh et al. recently demonstrated that a fatty acid load in mouse hepatocytes is reduced by macroautophagy(9).

## 2. Objectives

Investigations have explored the role of GLP-1 in FFA-induced pancreatic β cell death that the survivability is improved by stimulating GLP-1 receptor (10-12); However it is still unknown whether GLP-1 reduces β cells death by regulating macroautophagy.In this study, we will investigate the macroautophagy induced by FFA in INS-1 cells in the presence and absence ofLiraglutide. The results will provide a novel role for GLP-1 analogue in preventing or treating of T2D by confirming the role of GLP-1 on mediating autophagy in β cells.

## 3. Materials and Methods

### 3.1. Materials

Fetal bovine serum (FBS, Sigama), RPMI–1640 medium (Thermo Fisher Scientific, China), Palmitate (Sigma no. P-0500), Liraglutide (Novo Nordisk), 3-methyadenine (3-MA, sigma), MDC (sigma), Cell Counting Kit-8 (Japan-dojindo laboratories), Annexin V-FITC/PI (Baosai corporation of China ), BCA Protein Assay Kit (Bradford procedure), SDS-polyacrylamide gel electrophoresis, enhanced chemiluminescence (ECL) detection kit were obtained from GE healthcare (Buckinghamshire, UK), rabbit anti–light chain 3B (LC3B) antibody (Cell Signaling Technology company), β-actin antibody from Santa Cruz BiotechnologyInc, anti-rabbit secondary antibody (Jackson Immunoresearch Laboratories Inc. West Grove, PA, USA).

### 3.2. Cells

INS-1 rat insulinoma cells (purchased from ACTT)were grown in RPMI 1640 medium supplemented with 10% (v/v) fetal bovine serum (FBS) in a humidified atmosphere containing 95% air and 5% CO2.

### 3.3. FFA Preparation, Cell Treatment, and Lyses

100 mmol l–1 palmitate was prepared in 0.1 m NaOH at 70℃ and filtered. 5% (w/v) FFA-free BSA(Sigma no. A-6003) solution was prepared in double-distilled H2O and filtered (13). A 5mmol l–1 FFA/5% BSA (w/v) solution was prepared by mixing an appropriate amount of FFA to 5% BSA (w/v) in a 60 ℃ water bath. The above solution was then cooled to room temperature and diluted 1:5 in RPMI 1640 without FBS to a final concentration of 1 mm FFA/1% BSA (w/v).

### 3.4. CCK8 Viability Assay

Cell viability was assessed with Cell Counting Kit-8 (CCK8) (Dojindo Laboratories, Kumamoto, Japan) according to the manufacture's protocol. CCK8 is more sensitive than the 3-(4,5-dimethylthiazol-2-yl)-2, 5-diphenyltetrazolium bromide assay (14). INS-1 cells were treated with cck8 at 37°C for 1h. Absorbency was measured at 450 nm using a microplate reader.

### 3.5. Annexin V/ PI (PropidiumIodide) Staining

INS-1 cells were stained with both Annexin V and PI using the Annexin-V-FLUOS staining kit (Roche) in accordance with the manufacturer’s instructions. Briefly, cells plated on to 24- well plates at a density of 1.2×105 cells per cm2, were treated with lysosomal proteases or staurosporine and stained with incubation buffer containing AnnexinVand PI for 15 min at room temperature after removal of the medium. Apoptotic cells were imaged using a fluorescence microscope (Nikon Eclipse TE2000- U) with an excitation wavelength in the range of 450–500 nm and detection in the range of 515–565 nm. For quantification, nuclear staining was achieved by incubating the cells with Hoechst 33342 (Invitrogen) for 10 min at room temperature (15).

### 3.6. MDC Staining

To detect the autophagic vacuoles, cells were grown in the Lab-Tek Chambered no. 1.0 Borosilicate Coverglass System (NalgeNunc Intl., Rochester, NY). After treatment with PA for 24 h, the cells were stained with 50 um MDC in PBS at 37°C for 10 min. After washing with PBS, the cells were immediately analyzed under a fluorescence microscopy (355 nm excitation and 460 nm emission)(16).

### 3.7. Western Blot Analyses

Equal protein amounts (50 ug) were resolved by SDS-PAGE, transferred to nitrocellulose membranes, and immunoblotted with antibodies described above. After incubation with secondary antibody conjugated to horseradish peroxidase, the bands were detected with the enhanced chemiluminescence system. Immunoblots were scanned and quantified using Scion Image software.

### 3.8. Transmission Electron Microscopy (EM)

INS-1 cells were fixed with Karnovsky’s fixative solution [1% (v/v) paraformaldehyde, 2% (v/v) glutaraldehyde, 2mmol l–1 calcium chloride, and 100 mmol l–1 cacodylatebuffer (pH 7.4)] for 2 h and washed with cacodylate buffer. After postfixing with the fixative solution containing 1% (v/v)osmium tetroxide and 1.5% (v/v) potassium ferrocyanide for 1 h, the cells were dehydrated with 50–100% (v/v) alcohol and stained with en bloc in 0.5% (v/v) uranyl acetate. The cells were then embedded in Poly/Bed 812 resin (Pelco, Redding, CA) and polymerized, after which they were sectioned by Reichert Jung Ultracut S (Leica, Wetzetlar, Germany) and stained with uranyl acetate and lead citrate. The cells were observed and photographed under a transmission electron microscope (EM902A; Carl Zeiss MicroImaging GmbH, Germany) (16).

### 3.9. Statistics

Data are presented as mean±SE of at least three independent experiments. Statistical differences between the various groups were determined using the Student’s t test. P values less than 0.05 were considered statistically significant.

## 4. Results 

### 4.1. Liraglutide Enhances Survivability in INS-1 Cells

**Figure 1.**
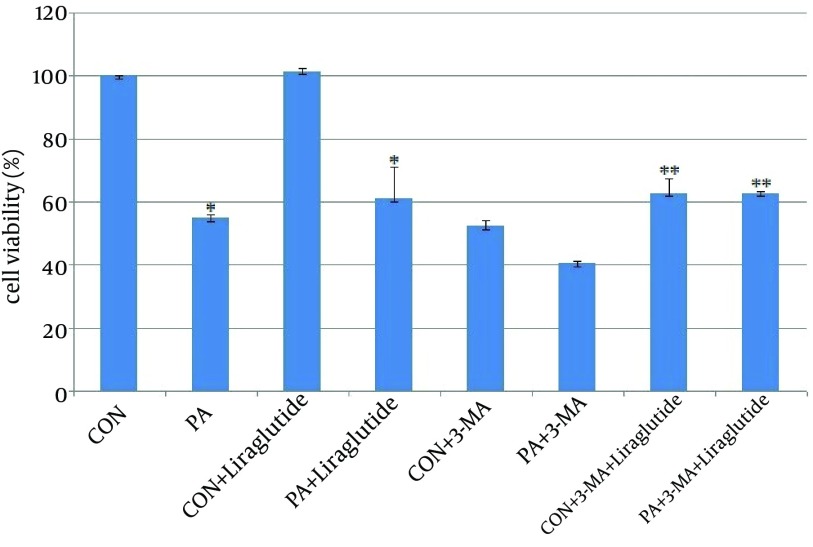
Liraglutide Improves Cell Viability in INS-1 Cells. INS-1 cells were treated with PA 0.5 mmol l–1 for 24h and then supplemented with Liraglutide 100 nmol l–1.INS-1 cells were pre-treated with 3-MA for 1h. Cell viability was measured by CCK8 assay.*, P <0.001 vs. viability of cells cultured in the CON (BSA 0.5%).**, P < 0.001vs. viability of cells cultured in the CON+3-MA.

We incubated INS-1 cells with 0.5 mmol l–1 PA in the presence of 5 mmol l–1 glucose for 24h. The viability of INS-1 cells was reduced dramatically with the treatment of PA by CCK8 assay. As shown in Figure 1, the INS-1 cells viability was significantly decreased to 55% in PA group compared with the control group (Figure 1). INS-1 cells were then treated and engorged with Liraglutide to determine if Liraglutide protected INS-1 cells from death. The survival of INS-1 cell with PA was improved to 85% after treating with liraglutide100 nmol l–1 for 24h (Figure 1). The results suggested that Lireglutide protects INS-1 cells from death induced by PA. 

As a classⅢ PI3K inhibitor, 3-MA significantly reduced INS-1 cells viability to 54% and 40% by treating with 3-MA alone and 3-MA plus with PA (Figure 1). Liraglutide improved the viability to 73% and 64% respectively, the percentage of which is lower than that of adding Liraglutide without pre-treated with 3-MA (Figure 1). The results demonstrated that inhibition of autophagy decreased INS-1 cells viability, and this effect could be strengthened by PA.

### 4.2. Liraglutide Inhibits FFA-Induced Apoptosis in INS-1 Cells 

**Figure 2.**
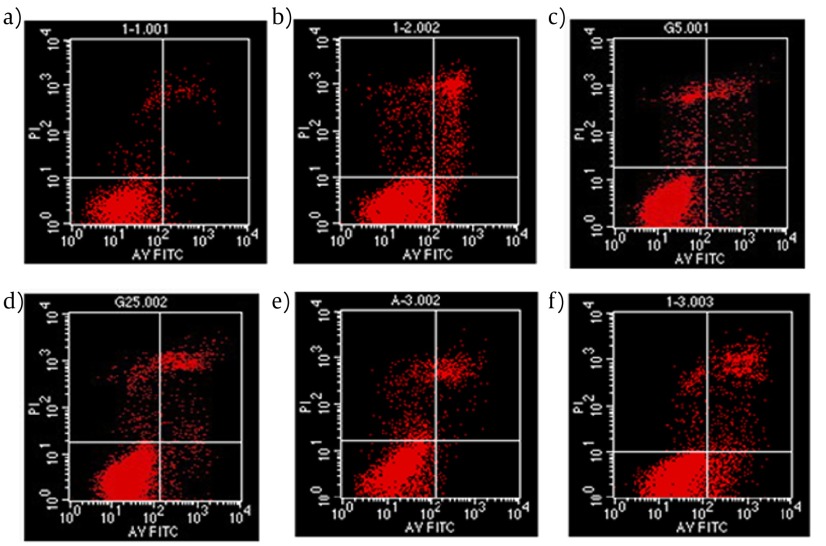
Liraglutide inhibits FFA-induced apoptosis in INS-1 cells. INS-1 cells were pre-treated with Liraglutide (100 nmol l–1) for 24h, then exposed to palmitate (PA, 0.5 mmol l–1) for 24h. The rate of apoptosis was determined by flow cytometric assay after Annexin V-FITC/PI staining. (a) control; (b) PA alone; (c) Liraglutide alone; (d) PA+Liraglutide; (e) 3-MA; (f) PA+3-MA;

An Annexin V-FITC/PI quantification assay demonstrated that PA-induced INS-1 cells were mediated by apoptosis. In our study, the apoptosis of INS-1 cells increased to 19% induced by PA compared with 5% in control group (Figure 2b). In addition, as shown in Figure 2d, apoptosis proportion was reduced to 13% suggesting a protective role of Liraglutide in INS-1 cells form PA-induced apoptosis. Autophagy inhibitor, 3-MA, produced an increased apoptosis to 10% in 3-MA group (Figure 2e) and 26% in PA+3-MA group (Figure 2f).

### 4.3. Liraglutide Inhibits PA-Induced Apoptosis by Stimulating Autophagy

**Figure 3.**
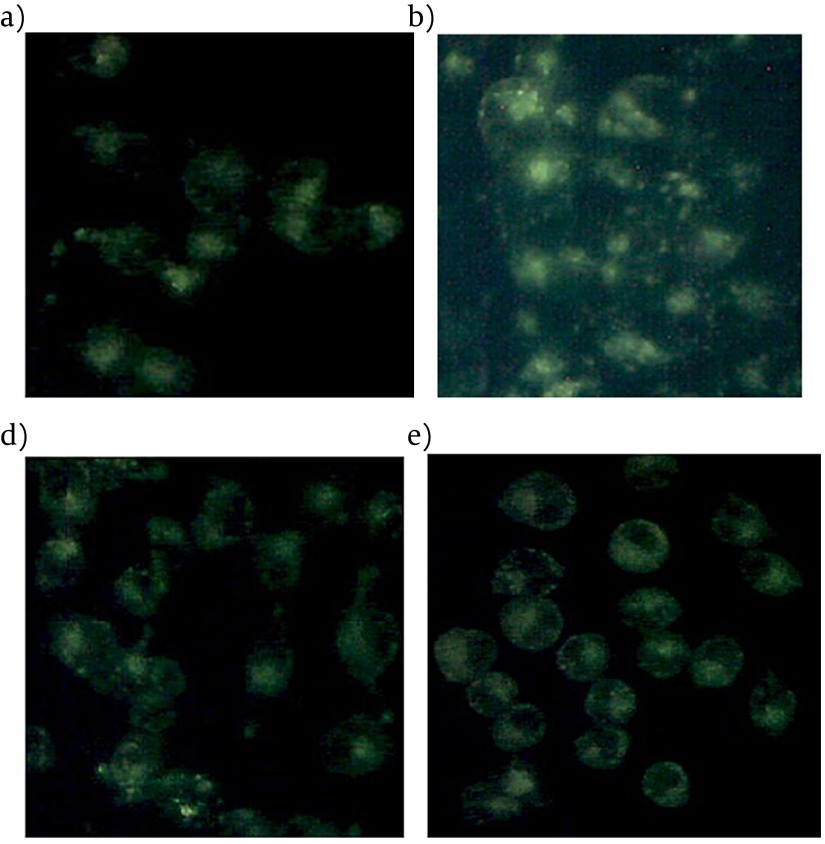
Liraglutide Induced Autophgy in INS-1 Cells Loaded with PA. The INS-1 cells treated with 0.5 mmol l–1 PA for indicated time durations were stained with 50 um MDC for 10 min. The fluorescent dots were observed under a fluorescence microscope. (a) control; (b) palmitate 0.5 mmol l–1; (c) Liraglutide 100 nmol l–1 ; (d) palmitate 0.5 mmol l–1 and Liraglutide 100 nmol l–1 .

When MDC, an autofluorescent compound used for the in vivo labeling of autophagic vacuoles, was applied to PA-treated INS-1 cells with Liraglutide, the proportion of cells with MDC stained dots was dramatically increased (Figure 3).

**Figure 4.**
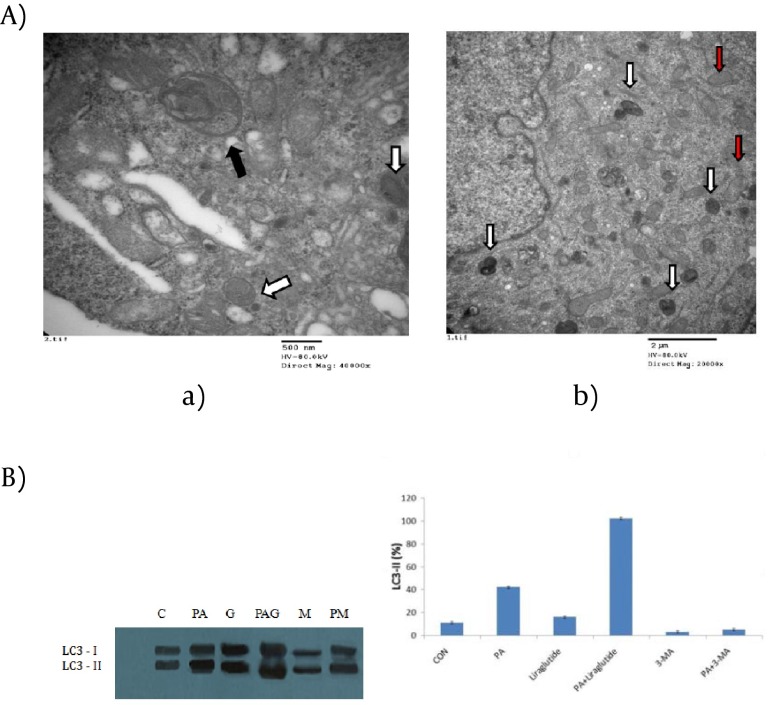
Liraglutide Stimulates Autophagy in INS-1 Cells with PA Treatment. (A) electron microscopy of INS-1 cells. (a) control; (b) palmitate+Liraglutide. Autophagosome (black arrowheads); autophagolysosome (white arrowheads); swelling mitochondria (red arrowheads). (B) Immunoblot for LC3 in palmitate acid and /or Liraglutide. C: control; PA: palmitate acid; G: Liraglutide; PAG: palmitate+Liraglutide; M: 3-MA; PM: palmitate acid+3-MA. The band intensity was determined using a one-dimensional image analysis program. The maximum intensity of LC3-Ⅱ was converted to 100%, and the relative intensities were calculated on the basis of maximum intensity. Data are expressed as the mean±SD values for three independent experiments. *, P <0.05 vs. LC3-Ⅱlevel of control group.

There was also an obvious increase of MDC stained dots in INS-1 cells following PA treatment alone for 24h, but relatively intact cellular structures could only be seen with Liraglutide treatment. No obviously increased MDC stained dots were showed in cells in the presence of Liraglutide alone. The results suggested that Liraglutide induced autophagy in INS-1 cells loaded with PA, and the induced autophagy was necessary to maintain the structure, mass and function of INS-1 cells. A typical autophagosome, double-limiting membrane, was detectable in the autophagosome (black arrowheads) and autophagolysosome (white arrowheads) (Figure 4A(a)) examined by EM. Ultrastructural images analysis showed the presence of double-membrane autophagic vesicles containing cell organelles in the cytoplasm of INS-1 cells. There was an increased number of autophagic vacuoles (AV), including autophagosomes and autophagolysosomes (AL), and also swelling mitochondrias in PA-treated INS-1 cells with Liraglutide. (Figure 4A(b)). As LC3B-Ⅱis the key protein associated with macrozutophagy, the conversion of LC3-Ⅰto LC3B-Ⅱwas examined. Liraglutide increased LC3B-Ⅱprotein levels in PA-induced INS-1 cells （Figure 4B）.

## 5. Discussion

Direct deleterious effects of FFAs on β cells are collectively termed "lipotoxicity"(17). PA elicited INS-1 cells death mainly through apoptosis. Results in our study showed decreased proliferation and increased apoptosis in INS-1 cells induced by PA coincidence with previous study of Sung-E Choi's (16). An improved INS-1 cells survival was observed following treatment with Liraglutide in FFA environment, suggesting that Liraglutide protected β cells from death. In addition, the ultrastructure analysis of PA-treated INS-1 cells clearly demonstrated the formation of double-membrane autophagosomes and single membrane autolysosomes with the treatment of Liraglutide. Conversion of LC3-Ⅰto LC3-Ⅱin FFA-induced INS-1 cells was significantly increased following the addition of Liraglutide. Such conversion was increased in presence of FFA alone with relatively weak efficiency (16). Our results demonstrated that Liraglutide was able to elicit autophagosome formation. Some debris in autolysosomes suggested that cytoplasmic organelles were functionally degraded by autophagy. All the data presented in this report suggested that FFA-induced INS-1 cells death was improved in the presence of Liraglutide by stimulated autophagy. 

It was previously proposed that β cells lipotoxicity was directly induced by PA at least in part via pathways involving ERs and reactive oxygen species (ROS)(17, 18). Lipotoxic ERs-mediated β cell dysfunction and apoptosis may be relevant in the development of T2D. Saturated and, to a lesser extent, unsaturated FFA trigger β cell ER stress. Physical ER stress occurs when high demand for protein load occurs, which is readily mitigated by UPR and is actually favorable to the β cells (19). But persistent ER stress results in rapid accumulation of unfolded proteins, which triggers β cell apoptosis (20). It is suggested that β cell survival in FFA was the consequence of the enhanced capacity to handle the UPR and thereby prevented INS-1 cell apoptosis from Lipotoxic ERs. In addition to ERs, pancreatic β cells were prone to oxidative stress, due to the fact that antioxidants such as superoxide dismutase (SOD), glutathione peroxidase and catalase were present at low levels in β cells (21). Oxidative stress can also lead to the accumulation of misfolded proteins. According to these published results, the protective role of Liraglutide observed in our study might be due to the degradation of misfolded proteins accumulation. 

Liraglutide, a long-acting GLP-1 analogue, is more efficient in protection of β cells than native GLP-1. Liraglutide inhibited cytokine-induced and FFA-induced apoptosis in islet cells in a dose-dependent manner, which has been concluded that liraglutidemight be useful for retaining β cell mass in both type 1 and type 2 diabetic patients (3). Liraglutide mediates its anti-apoptotic effects through GLP-1 receptor and its associated signaling cascade (3). Shimoda‘s results supported that liraglutide affected pancreatic β cell mass in diabetic conditions by directly stimulating cellular proliferation and could reduce triacylglycerol content in db/db mice (22). Several studies provided evidences of Liraglutide of ameliorating ERs. Liraglutideexerts its effects by inducing mitochondrial fusion, which prevented the onset of ERs induced by high-glucose (23). In addition, Liraglutide reduced and suppressed oxidative and ER stress by downregulating pro-apoptosis genes and those involved in lipid synthesis (22). The ultrastructure analysis of PA-treated INS-1 cells in our study clearly indicated the presence of induced autophagy. Not only did these results suggestedLiraglutide stimulated autophagy, but it also provided evidences as to why Liraglutide could improve INS-1 cell survival.

To investigate the role of autophagy in PA-induced INS-1 cell death, 3-MA, a specific inhibitor of class III PI3K was used in our research. An augment of PA-induced INS-1 cell death was shown following treatment of 3-MA, indicating a protective role of autophagy in PA environment. As an adaptive process responding to the metabolic stress, the activated autophagy finally results in degradation of intracellular protein and organelles (24). Some debris in autolysosomes suggested that cytoplasmic organelles were functionally degraded by autophagy (16). Thus, autophagy was responsible for removing the overload of unfolded and misfolded protein that exceeded the ER capacity (25). On the other hand, autophagy could also be implicated in cell death via apoptosis. It was possible that autophagy determined cell fate depending upon the kind and severity of stress (26, 27). In recent research, macro-autophagy has been identified as a mechanism for removal of fatty acid loads from hepatocytes (7). Studies by Singh et al. (9) considered autophagy targeting lipids inside the celllipoautpphagy. Sharma concluded that GLP-1 reduced the fat load in hepatocytes by inducing autophagy. Exendin-4 significantly increased the rate of autophagosome and autophagolysosome formation or autophagic flux (7). All the published results indicated that Liraglutide induced the level of autophagy to ameliorate FFA-induced cell death from different perspectives. Firstly, Liraglutide exerts its effect through controlling lipid synthesis, and the other mechanism was to remove already accumulated fat load in cells. These provided data consistently supported our observations that Autophagy has been involved in Liraglutide's protective effect.

In conclusion, our data showed that Liraglutide stimulated autophagy in PA-treated INS-1 cells, and induced autophagy played a protective role in PA-induced cell death. Autophagy exerts its effect in ameliorating ERs and removing fat load. Therapies that increase β cell resistance to FFA by Liraglutide, may have clinical application, as they prevent T2D or attenuate the progression of the disease.
